# Foot-and-Mouth Disease

**Published:** 1903-04

**Authors:** Austin Peters

**Affiliations:** Chief of Cattle Bureau of Massachusetts State Board of Agriculture


### FOOT-AND-MOUTH DISEASE IN MASSACHUSETTS.

By Austin Peters, M.R.C.V.S.,
CHIEF OF CATTLE BUREAU OF MASSACHUSETTS STATE BOARD OF AGRICULTURE.

(Concluded from page 143.)

Beside sending a letter to the inspectors of animals calling their attention to the existence of the disease, a letter was immediately sent to the Cattle Commissioners, or State Veterinarians, in all adjoining States except Rhode Island, notifying them that a disease existed in Massachusetts, the symptoms of which resembled foot-and-mouth disease, and as it appeared to be very contagious, they were advised to refuse all applications for permits from persons wishing to ship cattle from Massachusetts into their States. This practically gave every adjoining State an opportunity to quarantine against Massachusetts. No notification was sent to the Rhode Island Cattle Commission, as it was evident that there was as much reason to consider Rhode Island infected as Massachusetts.

The following order was approved by the Governor and Council, which acted as a quarantine against all neat cattle, sheep, or swine in Rhode Island :

November 18, 1902.

*To Transportation Companies and all Others Whom it May Concern :*

By virtue of the power and authority vested by law in the Cattle Bureau of the State Board of Agriculture under the provisions of Chapter XC. of the revised laws and Chapter CXVI. of the Acts of 1902, it is hereby ordered that no neat cattle, sheep, or swine be brought into Massachusetts from the State of Rhode Island until further notice, because of the prevalence of a disorder among cattle, of an apparently contagious character, in the vicinity of Cumberland, R. I., which resembles foot-and-mouth disease.

Inspectors of animals in the cities and towns bordering on the Rhode Island line will see that this order is enforced.

Austin Peters,
Chief of Cattle Bureau.

Approved in Council November 19, 1902.

E. F. Hamlin,
Executive Secretary.

As the situation seemed more threatening, on November 26th an order was adopted by the Governor and Council stopping the market held at Brighton every week until further notice. This order is still held in force.

COMMONWEALTH OF MASSACHUSETTS,
CATTLE BUREAU OF THE STATE BOARD OF AGRICULTURE,
STATE HOUSE.

BOSTON, November 26, 1902.

*To the Brighton Stockyards Company and all Others Whom it May Concern:*

By virtue of the power and authority vested by law in the Cattle Bureau of the State Board of Agriculture, under the provisions of Chapter XC. of the revised laws and Chapter CXVI. of the Acts of 1902, you are hereby notified that foot-and-mouth disease, which is a contagious disease and is so recognized under the laws of this Commonwealth, exists among cattle, sheep, and swine in some sections of this State, and also in certain localities in Rhode Island.

You are hereby further notified that in order to prevent the spread of this disease this bureau has issued the following order, to continue until the public safety will allow of its being revoked by the Chief of the Cattle Bureau, with the sanction of the Governor and Executive Council:

1. The public market for milch cows, working oxen, store cattle, store sheep, or store swine, held at Brighton each week, is hereby ordered to be discontinued. Beef cattle, sheep, or swine under control of the United States Bureau of Animal Industry, Department of Agriculture, intended for export, are not included in this order. Beef cattle, sheep, swine, or veal calves, except animals from Rhode Island, may be shipped to the stockyards at Brighton, Watertown, or Somerville, provided they are for immediate slaughter.

2. Transportation companies bringing cattle, sheep, or swine into Massachusetts for the stockyards at Brighton, Watertown, and Somerville, are hereby ordered not to accept shipments of milch cows, working oxen, or store cattle, or store sheep or store pigs, for these yards; and are further reminded that no neat cattle are to be received for shipment to other points unless accompanied by the usual permit required under the order of April 23, 1902. The order of November 19, 1902, forbids transportation companies to receive any neat cattle, sheep, or swine from the State of Rhode Island.

3. Transportation companies are also to clean all manure and litter from cars used in local animal transportation after unloading neat cattle, sheep, or swine, depositing it where it will not come in contact with neat cattle, sheep, or swine; and after thoroughly scraping out the car, it must be washed on the floor and sides with a 4 per cent. solution of chloride of lime.

4. Any person violating the provisions of this order will be punished as provided in Section 29 of Chapter XC. of the revised laws.

This order shall take effect upon the day following its approval by the Governor and Council.

AUSTIN PETERS,
Chief of Cattle Bureau.

Approved in Council November 26, 1902.

E. F. HAMLIN,
Executive Secretary.

December 1st a special meeting of the Governor and Council was held, giving the Chief of the Cattle Bureau still further power to cope with the disease.

COMMONWEALTH OF MASSACHUSETTS,
CATTLE BUREAU OF THE STATE BOARD OF AGRICULTURE.
STATE HOUSE.

BOSTON, December 1, 1902.

*To all Persons Whom it May Concern:*

By virtue of the power and authority vested by law in the Cattle Bureau of the State Board of Agriculture, under the provisions of Chapter XC. of the revised laws and Chapter CXVI. of the Acts of 1902, you are hereby notified that foot-and-mouth disease, which is a contagious disease and is so recognized by the laws of the Commonwealth, exists to an alarming extent among cattle, sheep, and swine in some sections of this State. You are hereby further notified that in order to prevent its spread this bureau has issued the following order, to continue until revoked by the Chief of the Cattle Bureau:

1. All neat cattle, sheep, and swine upon infected premises are to remain in quarantine until such time as the Chief of the Cattle Bureau decides that it is proper to release them, and no neat cattle, sheep, or swine are to be brought upon or removed from such premises without his permission, upon any pretext whatsoever. The disposal of the products or manure of such animals, or litter, hay, straw, utensils, and all other material are subject to the orders of the Chief of the Cattle Bureau.

2. All persons not having business upon premises deemed by the Chief of the Cattle Bureau to be infected with foot-and-mouth disease are hereby forbidden to trespass thereon.

3. No auctions or public sales of neat cattle, sheep, or swine shall be held in localities deemed by the Chief of the Cattle Bureau to be infected, without his permission.

4. All persons are forbidden to transport any neat cattle, sheep, or swine over the public highway, or to turn the same upon any unfenced land in such city or town wherein the foot-and-mouth disease exists, and after notice thereof has been given by the Chief of the Cattle Bureau, without his special permission so to do.

5. All persons are forbidden to tamper with or disfigure any notices posted by order of the Chief of the Cattle Bureau, subject to the penalty of the law.

This order takes effect upon its approval.

AUSTIN PETERS,
Chief of Cattle Bureau.

Approved in Council December 1, 1902.
E. F. HAMLIN,
Executive Secretary.

A great deal of the work for the first two weeks of the outbreak was preliminary to getting matters in shape to co-operate with the United States authorities. Notices of various kinds were printed and sent out, and the necessary steps to be taken became apparent as different phases in connection with the outbreak developed.

The following notice was sent out for the benefit of all interested persons, to be posted in public places and given to interested individuals by the inspectors of animals in infected localities:

COMMONWEALTH OF MASSACHUSETTS,
CATTLE BUREAU OF THE STATE BOARD OF AGRICULTURE.

NOTICE.

*Whereas*, a contagious disease of cattle, sheep, and swine known as foot-and-mouth disease, and recognized as such by the laws of this Commonwealth, has recently made its appearance in certain localities in this State, and has spread to an alarming extent in certain places. The attention of all persons is called to the following provisions of the law:

Section 11, Chapter XC. of the revised laws, and Chapter CXVI., Acts of 1902.

The board of health of a city or town, any member or agent thereof, or any other person who has knowledge of or reason to suspect the existence of any contagious disease among any domestic animals in this Commonwealth, or that any domestic animal is affected with such contagious disease, whether such knowledge is obtained by personal examination or otherwise, shall immediately give notice thereof in writing to the Chief of the Cattle Bureau of the State Board of Agriculture, or to any of its agents or inspectors. Whoever fails to give such notice shall be punished by a fine, not exceeding one hundred dollars.

Foot-and-mouth disease is characterized by slobbering, or drooling, smacking of the lips, and lameness. Upon a closer examination a vesicular eruption will be found inside the lips, on the dental pad, in the mouth, on the tongue, between or around the digits, and upon the udder and teats. Later the tops of the blisters peel off, leaving superficial ulcerating sores.

It is highly contagious, and may be spread by healthy animals walking upon the highways where contaminated creatures have been driven, by the use of stock-cars which have not been disinfected, by utensils, and by attendants.

All persons are warned against going from infected premises to those where healthy animals are kept.

The milk from diseased cows is unsafe for food for man or cloven-footed animals, unless cooked or properly sterilized.

This malady is a menace to the public health and to commercial prosperity, and all good citizens are requested to co-operate with Federal and State authorities in assisting in its eradication, by reporting all cases that come to their knowledge, and by keeping away from infected premises.

AUSTIN PETERS,
Chief of Cattle Bureau.

STATE HOUSE, BOSTON, December 1, 1902.

Following this was another letter of instruction to inspectors, telling them what they were expected to do within the next few days, and calling for a rapid inspection of all the neat cattle, sheep, and swine in every city or town east of the Connecticut river. Inspectors of animals of the border towns between Massachusetts and Rhode Island, Connecticut or Vermont, received cards to be tacked up on trees on the highways inside the Massachusetts line, forbidding moving cattle, sheep, other ruminants, or swine across the boundaries of the State in either direction. Placards were also printed and distributed, to be tacked on buildings where the disease existed, forbidding the removal of any neat cattle, sheep, or swine from the premises, or the introduction of any new animals until the quarantine was officially removed by the Chief of the Cattle Bureau. This placard also forbid trespassing on the quarantined premises for fear that persons might carry the infection from one place to another upon the hands, clothing, or shoes.

Other placards were sent out for inspectors of animals to post, forbidding auction or public sales of neat cattle, sheep, other ruminants, or swine in the counties of Essex, Middlesex, Suffolk, Plymouth,

Norfolk, Bristol, or Worcester, unless the person wishing to hold such sale had a permit from the Chief of the Cattle Bureau.

Placards were also sent out, to be tacked up in conspicuous places in towns infected with foot-and-mouth disease, forbidding all persons to drive or transport any neat cattle, sheep, other ruminants, or swine over any public highway in said town without a permit signed by the Chief of the Cattle Bureau or his authorized representative, or to turn out any such animals on any unfenced lands until such time as it was declared by the Chief of the Cattle Bureau to be safe for the cattle traffic to be resumed.

All this means a great deal of work, but it can be seen that, after taking all these measures, everything was prepared to proceed with the more active work of eradication.

Within a few days of the discovery of foot-and-mouth disease in Dedham, quarantines on infected herds commenced to pour in from different directions. As soon as the disease was found in a town, placards, already referred to, were sent to the Inspector of Animals to tack up, prohibiting the movement of cloven-footed animals in the public highways or turning the same upon any unfenced lands.

The following is a list of towns declared to be infected which were placarded in this way, giving the dates when the placards were sent to the Inspectors of Animals:

| | | | | | |
|---|---|---|---|---|---|
| Acton | By ex. | 12-6 | Maynard | By ex. | 12-6 |
| Attleborough | " | 12-6 | Marlborough | " | 12-8 |
| Andover | " | 12-6 | Methuen | " | 12-6 |
| Arlington | " | 12-23 | Natick | " | 12-9 |
| Billerica | " | 12-6 | Needham | " | 12-8 |
| Boxborough | " | 12-6 | North Attleborough | " | 12-6 |
| Bridgewater | " | 12-6 | North Reading | " | 12-8 |
| Braintree | " | 12-12 | North Andover | " | 12-6 |
| Concord | " | 12-6 | Pepperell | " | 12-6 |
| Chelmsford | " | 12-6 | Quincy | " | 12-8 |
| Chelsea | " | 12-6 | Revere | " | 12-6 |
| Carlisle | " | 12-6 | Stow | " | 12-6 |
| Dedham | " | 12-8 | Sudbury | " | 12-6 |
| Dover | " | 12-29 | Southborough | " | 12-6 |
| Everett | " | 12-6 | Sharon | " | 12-8 |
| Framingham | " | 12-12 | Wayland | " | 12-6 |
| Grafton | " | 12-10 | Weston | " | 12-6 |
| Harvard | " | 12-6 | Westford | " | 12-6 |
| Hudson | " | 12-6 | Westborough | " | 12-6 |
| Hatfield | " | 12-23 | West Bridgewater | " | 12-8 |
| Lincoln | " | 12-8 | Westwood | " | 12-23 |
| Littleton | " | 12-6 | Watertown | " | 12-23 |
| Lawrence | " | 12-23 | | | |

6 green cards, auction forbidden, etc.,
6 white " posters for buildings infected, etc.,
40 yellow " towns declared infected, etc.,
} were sent to the Inspectors of Animals in each town.

A great deal of the trouble northwest of Boston has been due to an auction, November 17th, at which one hundred head of cattle were sold, and wherever they went foot-and-mouth disease was carried. This auction scattered it to Barre, Mass., and Chester, Vt., as well as to many of the towns adjoining Acton, where the auction was held.

The towns of North Attleborough and Attleborough were infected by cattle brought across the line from Rhode Island. The appearance of the disease in Methuen and other towns adjoining Lawrence was probably due to cattle taken to Lawrence from the Brighton market. The herds which were infected in Bridgewater, West Bridgewater, Quincy, and Braintree received cattle from droves taken from Brighton. How the single herd that had foot-and-mouth disease in Danvers became contaminated it is very difficult to say, as there is no report of any other cases in that immediate locality. The owner of the cattle is a market gardener, and it is possible he may have taken produce to Chelsea and, when he came home, used some of the boxes which had been lying around in Chelsea to feed the cows from.

It is a difficult matter to say, positively, just where the disease came from, but as nearly as can be ascertained it appeared as long ago as August in the neighborhood in Chelsea known as Prattsville, and as Chelsea is next to East Boston, where the foreign shipping comes in, it is not unlikely that the infection was brought over on a foreign steamer in hay or in straw used for packing merchandise, and in some way was carried to one of the places in this locality, and other herds near by were contaminated. As the lines of Chelsea, Revere, and Everett converge at this point, cases have been found in all three places, but the owners of the cattle where the disease existed do not live very far apart.

It is said that foot-and-mouth disease assumes a milder form in summer than in winter, and the herds first infected probably had a form of the disease that was not particularly virulent, and nothing was thought of it. It is very clear that the cow that carried the trouble to Rhode Island went directly from Chelsea to Brighton, and thence to Cumberland. Other cattle from the infected neighborhood found their way later to the Brighton market, until the yards became infected, and in November it was carried in various directions by different animals. The disease was taken to Acton by two or three cows from Brighton, which contaminated the hundred head sold there at auction November 17th.

The inspector of animals of Malden is a veterinarian. He was called in a professional capacity the last of August to see a herd of cattle in Revere which he says showed symptoms of foot-and-mouth disease; but he supposed that it did not exist in the United States, and thought it was probably something resembling it. He was about to go to Minneapolis to a meeting of the American Veterinary Medical Association, held there the first week in September. Before going he says he telephoned to the Inspector of Animals in Chelsea, thinking at the time that the herd which he had seen was in Chelsea and not in Revere, and supposed that he had complied with the law requiring anyone to report a disease of an apparently contagious character to the local inspector of animals. As the herd happened to be in Revere, the Chelsea inspector was not the man to look after it, and he says that he does not even remember receiving any notification. The Malden veterinarian returned home after the meeting, and never gave the subject a second thought until the outbreak was called to the public attention in the morning newspapers the day after Thanksgiving. It is therefore apparent that foot-and-mouth disease existed in the locality of Prattsville as far back as the last of August.

As the Inspector of Animals of Chelsea and Everett is a veterinarian, and the Inspector of Animals for the town of Revere is also a veterinarian, it seems strange that when they made the annual inspection of the neat cattle in these towns, which was ordered by the Chief of the Cattle Bureau to commence October 1st and be completed November 15th, no suspicious cases were observed by these inspectors; but it is not unlikely that when they made their rounds the cases which had been ailing had recovered to such an extent that symptoms of the disease escaped their observation. If the disease had been reported when it first appeared in the neighborhood of Prattsville it might easily have been held in check there and a great financial loss saved to the community. [1]

The following table shows the number of herds quarantined up to December 31st and the disposition made of them:

[1] In fact, the owner of one of the infected herds in Chelsea says that he had the Inspector of Animals examine his cattle in a professional capacity about the middle of September, at which time the inspector called the disease foot-rot, and did not recognize its character. If the above statement is true, the Inspector of Animals made a grave error in not reporting it, as he should have done, to the Cattle Bureau at that time.

### Statistics of Foot-and-Mouth Disease in Massachusetts, December 31, 1902.

Showing the towns in which the disease has appeared or been suspected, the number of herds and number of animals quarantined, number released, number killed, etc., to date.

### NEAT CATTLE.

| Towns. | Quarantined. | | Released. | | Killed by U. S. Government. | | Apprais'd value. | Per cent. paid. |
|---|---|---|---|---|---|---|---|---|
| | Herds. | Cattle. | Herds. | Cattle. | Herds. | Cattle. | | |
| Abington, | 1 | 21 | 1 | 21 | | | | |
| Acton, | 11 | 172 | ... | ... | 8 | 129 | $4,625.00 | $4,132.50 |
| Andover, | 1 | 47 | | | | | | |
| Arlington, | 1 | 13 | ... | ... | 1 | (13)[1] | | |
| Attleborough, | 2 | 48 | ... | ... | 2 | 47 | 1,895.00 | 1,326.50 |
| Auburndale,[2] | ... | ... | ... | ... | 1 | 1 | 55.00 | 38.50 |
| Avon, | 1 | 4 | 1 | 4 | | | | |
| Barre, | 2 | 30 | ... | ... | 2 | 37 | 1,530.00 | 1,071.00 |
| Belchertown, | 1 | 1 | 1 | 1 | | | | |
| Billerica, | 1 | 46 | ... | ... | 1 | 46 | 2,415.00 | 1,690.50 |
| Boston, | 2 | 18 | 1 | 8 | 1 | 10 | 525.00 | 367.50 |
| Boxborough, | 2 | 43 | | | | | | |
| Boxford, | 1 | 6 | | | | | | |
| Braintree, | 5 | 95 | 3 | 60 | 2 | 36 | 1,384.00 | 968.80 |
| Bridgewater, | 2 | 21 | ... | ... | 1 | 19 | 840.00 | 588.00 |
| Brockton, | 4 | 84 | 4 | 84 | | | | |
| Burlington, | 3 | 39 | | | | | | |
| Carlisle, | 5 | 64 | ... | ... | 4 | 60 | 2,665.00 | 1,865.50 |
| Chelmsford, | 2 | 72 | ... | ... | 2 | 59 | 2,385.00 | 1,997.50 |
| Chelsea, | 4 | 27 | | | | | | |
| Cohasset, | 1 | 2 | ... | ... | 1 | 2 | ......... | 80.00 |
| Concord, | 17 | 336 | 2 | 51 | 12 | 275 | 13,718.50 | 9,602.95 |
| Danvers, | 1 | 7 | ... | ... | 1 | 7 | 315.00 | 220.50 |
| Dedham, | 3 | 64 | ... | ... | 3 | 26 | 1,198.00 | 838.60 |
| Dover, | 1 | 24 | | | | | | |
| East Bridgewater, | 3 | 52 | 3 | 52 | | | | |
| Easton, | 1 | 7 | 1 | 7 | | | | |
| Everett, | 1 | 6 | | | | | | |
| Framingham, | 2 | 56 | ... | ... | 2 | 55 | 2,965.00 | 2,075.50 |
| Gloucester, | 1 | 1 | 1 | 1 | | | | |
| Grafton, | 1 | 10 | ... | ... | 1 | 10 | 485.00 | 339.50 |
| Hanover, | 1 | 14 | 1 | 14 | | | | |
| Harvard. | 1 | 6 | ... | ... | 1 | 5 | ......... | 125.00 |
| Hatfield, | 1 | 50 | | | | | | |
| Hingham, | 1 | 17 | 1 | 17 | | | | |
| Lawrence, | 1 | 5 | ... | ... | 1 | 5 | 260.00 | 182.00 |
| Leominster, | 1 | 1 | | | | | | |
| Lexington, | 1 | 1 | 1 | 1 | | | | |
| Lincoln, | 4 | 140 | 1 | 50 | 2 | 96 | 4,705.00 | 3,293.50 |
| Littleton, | 10 | 172 | 1 | 17 | 1 | 30 | 1,020.00 | 714.00 |
| Lowell, | 1 | 1 | | | | | | |
| Marlborough, | 3 | 49 | ... | ... | 3 | 48 | 2,414.00 | 1,689.80 |
| Marshfield, | 1 | 5 | 1 | 5 | | | | |
| Methuen, | 7 | 183 | ... | ... | 2 | 25 | 1,269.00 | 888.30 |
| Middleborough, | 2 | 14 | 2 | 14 | | | | |
| Milton, | 1 | 2 | | | | | | |
| Natick, | 1 | 16 | ... | ... | 1 | 14 | 750.00 | 525.00 |
| Needham, | 2 | 32 | ... | ... | 1 | 29 | 1,553.00 | 1,087.10 |
| North Andover, | 2 | 85 | ... | ... | 2 | 87 | 3,990.00 | 2,793.00 |
| North Attleborough, | 1 | 5 | | | | | | |
| Northborough, | 1 | 3 | 1 | 3 | | | | |
| North Reading, | 1 | 25 | 1 | 25 | | | | |
| Pepperell, | 3 | 8 | ... | ... | 1 | 2 | 55.00 | 38.50 |
| Quincy, | 9 | 112 | 6 | 82 | 3 | 31 | 1,455.00 | 1,018.50 |
| Randolph, | 1 | 14 | 1 | 14 | | | | |
| Raynham, | 1 | 1 | ... | ... | 1 | 3 | 125.00 | 87.50 |
| Revere, | 1 | 32 | | | | | | |
| Saugus, | 1 | 1 | 1 | 1 | | | | |
| Sharon, | 2 | 39 | | | | | | |
| Somerville, | 2 | 9 | | | | | | |

[1] Killed, as owned by Hayden, of Watertown.
[2] Probably included in a quarantine in Sudbury.

| Towns. | Quarantined. | | Released. | | Killed by U. S. Government. | | Apprais'd value. | Per cent. paid. |
|---|---|---|---|---|---|---|---|---|
| | Herds. | Cattle. | Herds. | Cattle. | Herds. | Cattle. | | |
| Southborough, | 5 | 164 | 1 | 1 | 4 | 166 | $8,625.00 | $6,037.50 |
| Stow, | 1 | 31 | ... | ... | 1 | 30 | 1,343.00 | 940.10 |
| Sudbury, | 3 | 61 | ... | ... | 1 | 1 | 50.00 | 35.00 |
| Taunton, | 4 | 83 | 3 | 68 | | | | |
| Walpole, | 1 | 4 | | | | | | |
| Watertown, | 2 | 44 | ... | ... | 2 | 58[1] | 2,785.00 | 1,974.50 |
| Wayland, | 2 | 29 | ... | ... | 1 | 12 | 1,029.00 | 720.30 |
| Wellesley, | 1 | 15 | 1 | 15 | | | | |
| Westborough, | 9 | 257 | 1 | 20 | 8 | 215 | 9,885.00 | 6,919.50 |
| West Bridgewater, | 6 | 104 | ... | ... | 4 | 97 | 4,763.00 | 3,334.10 |
| Westford, | 5 | 60 | 1 | 7 | 3 | 52 | 2,069.00 | 1,448 30 |
| Weston, | 3 | 72 | ... | ... | 2 | 23 | 1,422.00 | 995.40 |
| Westwood, | 1 | 85 | | | | | | |
| Weymouth, | 1 | 7 | 1 | 7 | | | | |
| Whitman, | 3 | 80 | 3 | 80 | | | | |
| Total, | 194 | 3554 | 47 | 730 | 90 | 1848 | $86,567.50 | $62,050.25 |

144 swine and 42 sheep are included in the above valuation, the special appraisal not being given.

In addition the following animals have been included in the above quarantines, some of which have been affected:

| | | | |
|---|---|---|---|
| Swine . . . . . | 815 | Goats . . . . . | 4 |
| Sheep . . . . . | 122 | Horse . . . . . | 1 |
| | | Total . . . | 942 |

SUMMARY.

| | |
|---|---|
| Total number neat cattle quarantined . . . . . . . . . | 3554 |
| " " other animals quarantined . . . . . . . . | 942 |
| Grand total . . . . . . . . . | 4496 |
| Total number cattle released from quarantine as not affected . . | 730 |

NOTE.—The two right-hand columns in the above table are subject to some slight changes, as complete returns have not been made. Where the appraised value is not given and the price paid appears in the extreme right-hand column, the cattle were bought outright.

In addition to the expense to the United States Government of killing and paying for the diseased animals is the expense to the government of a large number of inspectors detailed on this special work; also the extra cost to the State of Massachusetts for the employment of agents of the Cattle Bureau of the State Board of Agriculture and the disinfection of buildings, to say nothing of the commercial loss to the port of Boston. Another item of expense to the State will be the quarantine expenses. Section 21 of the revised laws provides as follows:

"Section 21. If animals have been quarantined, collected, or isolated upon the premises of the owner or of the person in possession of them at the time such quarantine is imposed, the expense thereof shall be paid by such owner or person; but if specific animals have

[1] This number includes 13 quarantined in Arlington.

been quarantined or isolated, under the provisions of Section 5 or Section 19, for more than ten days upon such premises as suspected of being affected with a contagious disease, and the owner is forbidden to sell any of the product thereof for food, or if animals have been quarantined, collected, or isolated on any premises other than those of such owner or person in possession thereof, the expense of such quarantine shall be paid by the Commonwealth."

The cattle owners will be notified of this provision of the law, and will also be furnished with the necessary blanks upon which to make their claim for the expense of quarantine. These claims will probably be sufficiently heavy to make it necessary for the Legislature to make a special appropriation to meet them, as it is doubtful if enough of the appropriation made for the work of the Cattle Bureau of the State Board of Agriculture during 1902 will be left to meet all the expenses incurred as the result of the outbreak of foot-and-mouth disease.

Besides the losses and expenses already spoken of it must not be forgotten that the loss to the owners of the animals which have been killed will not end with the killing of the animals, as barns where the disease has existed will have to be disinfected, and will then have to be kept empty for several weeks before new cattle can be introduced, and none of the forage on these infected premises ought to be removed for some time to come. This means that a number of farmers will for the next month or two be entirely deprived of their chief sources of income.

With the stringent measures that have been taken for the suppression of the disease the prospects are that it will soon be eradicated. It is to be hoped that in the course of a few weeks more it will be practically a thing of the past, although it may be necessary to keep up a system of inspection in some localities and certain restrictions on the movements of cattle for some time longer than this. As soon as the disease is eradicated the Brighton market can be re-opened and the cattle business resume its former conditions.

At the time of the visit of Dr. Theobald Smith and the Chief of the Cattle Bureau of the State Board of Agriculture to the infected herd in Dedham, November 16th, Dr. Smith carried some glass pipettes with him. Serum from a vesicle on a cow's udder was drawn into the pipettes after pricking the vesicle with a needle, and the ends sealed in a spirit lamp. These were placed in an ice-chest in Dr. Smith's laboratory, where they remained until the following Friday.

Mr. C. A. Dennen, agent of the Cattle Bureau at Brighton, sent two cows over to Bussey Institution, which were bought November 19th; also two small pigs, six or eight weeks old. The cows were inoculated November 21st by scarifying the gum under the upper lip with a lancet and rubbing in some of the lymph. One of the pigs was also inoculated in the mouth and between the toes on one fore foot; the other pig was kept to be fed upon the milk of the cows. These cows, after two or three days, developed typical lesions of foot-and-mouth disease in their mouths; one cow also showed very slight signs between the divisions of the fore foot. Neither cow ever showed any eruptions on the udder. They both had a very mild form of the disease, and in two or three weeks seemed to have fully recovered. December 11th the cows were killed, post-mortem only showing cicatrices in the mouths of both where the ulcers had been. In one there were a few ulcers still unhealed. Unfortunately the pig that was inoculated died a couple of days later. Post-mortem made by Dr. Smith showed it to have congestion of the lungs. As the pigs had probably been kept in a warm place and then put in a cold barn, it is to be supposed that the one that died must have taken cold. The other pig was fed the milk from the cows until he was killed, December 11th, but never at any time showed symptoms of the disease.

These experiments were undertaken to furnish additional proof, if any was needed, that the cows seen at Dedham were suffering from foot-and-mouth disease, and when the diagnosis was confirmed by Dr. Pearson and Dr. Law on their arrival, these experiments were advanced far enough to still further strengthen the diagnosis of foot-and-mouth disease.

